# Nicotinamide mononucleotide: a potential effective natural compound against insulin resistance

**DOI:** 10.1038/s41392-021-00723-z

**Published:** 2021-08-19

**Authors:** Julian Roos, Julia Zinngrebe, Pamela Fischer-Posovszky

**Affiliations:** Department of Pediatrics and Adolescent Medicine, Ulm University Medical Center, Ulm, Germany

**Keywords:** Endocrine system and metabolic diseases, Translational research, Molecular medicine

Recently, a study published in Science by Yoshino et al.^1^ reported on a randomized, placebo-controlled, double-blind clinical trial to examine the effects of a 10-week nicotinamide mononucleotide (NMN) administration on human metabolism in 25 postmenopausal overweight or obese women with prediabetes. It revealed positive effects of NMN on insulin sensitivity, insulin signalling, and tissue remodelling in skeletal muscle.

**Fig. 1:**
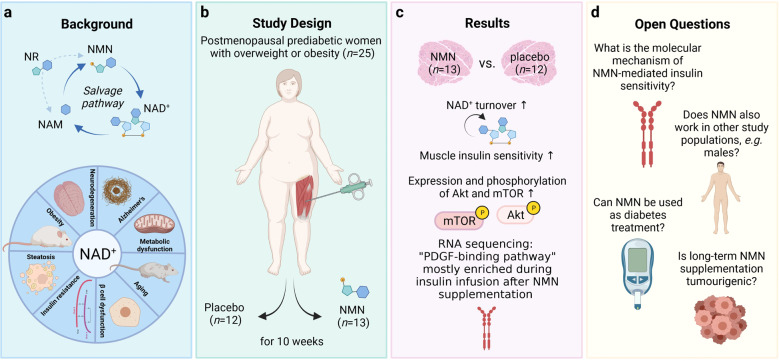
**a** In a salvage pathway nicotinamide ribose (NR) and/or nicotinamide (NAM) are converted to nicotinamide mononucleotide (NMN), which is the precursor of nicotinamide adenine dinucleotide (NAD^+^). NAD^+^ is involved in a spectrum of diseases. **b** Postmenopausal prediabetic women with overweight or obesity were treated with NMN or placebo for 10 weeks and muscle biopsies revealed, **c** increased muscle NAD^+^ turnover, insulin sensitivity, phosphorylation of mTOR and Akt, and a gene expression involving the PDGF-binding pathway in NMN-treated participants as compared to control. **d** Open questions, however, remain to fully assess the mechanism of NMN in mediating muscle insulin sensitivity. Furthermore, potential long-term side effects should be considered. The figure was generated with Biorender.com.

Nicotinamide (NAM) and nicotinamide ribose (NR) are converted to NMN, which is the precursor of nicotinamide adenine dinucleotide (NAD^+^), a co-substrate of NAD^+^-dependent enzymes essential for biological processes as important as redox homeostasis, gene expression, RNA processing, genomic stability, immunity and inflammation, and energy metabolism (reviewed by Xie et al.^2^). NMN is the main source of the salvage pathway to generate NAD^+^ (Fig. 1). In addition, mammalian cells generate NAD^+^ de novo via the Kynurenine and the Preiss-Handler pathways.^2^ Approximately 95% of circulating NMN is released by the liver. In rodents, NMN was reported to slow down aging and to prevent age-related disorders ranging from Alzheimer’s to diabetes to metabolic dysfunction^2^ (see Fig. 1). This is why NMN has become the new hope in the search for longevity, the “fountain of youth”. Thus, oral NMN supplementation became quite popular during the last decade, especially in the US. The study by Yoshino et al., however, is the first to deliver evidence of positive metabolic and insulin-sensitizing effects of daily NMN supplementation in humans.

Type 2 diabetes mellitus (T2DM) is one of the most serious health crises of our time with a prognosed prevalence of above 50% for 2045. Although there are 10 different US Food and Drug Administration (FDA)-approved classes of drugs for the treatment of T2DM and more than 7000 clinical trials are currently investigating new drug candidates, there is still an enormous need to develop effective medications with minor side effects.^3^

T2DM is a consequence of systemic insulin resistance with attenuated biological responses to normal or elevated insulin levels and subsequently impaired insulin-mediated glucose disposal. To maintain normal blood glucose levels, the pancreatic β-cells secrete more insulin resulting in compensatory hyperinsulinemia until they reach their maximal production capacity.^3^ Insulin resistance is caused by defects in signal transduction such as decreased insulin receptor expression and kinase activity, diminished expression and phosphorylation of insulin receptor substrate 1 (IRS-1), reduced phosphatidylinositol 3 (PI3)-kinase activity, and less glucose transporter type 4 (GLUT4) expression and translocation.^4^ Insulin stimulates glucose deposition and glycogen synthesis in adipose tissue, muscle and liver, and it inhibits gluconeogenesis and ketone body production in the liver. The musculature is the major site for whole-body insulin-dependent glucose uptake and therefore most relevant for glucose clearance.^4^

NAD^+^ is one of the most abundant metabolites and plays an essential role as co-substrate for various enzymes.^2^ Among the NAD^+^-dependent enzymes are sirtuins, a family of class III histone deacetylases mediating post-translational modifications of again other metabolic enzymes. Thereby, NAD^+^ availability modulates the citric acid cycle, cytosolic glycolysis, gluconeogenesis, glycogen metabolism, and mitochondrial fatty acid oxidation.^2^ An imbalance of NAD^+^ homeostasis is associated with mitochondrial dysfunction, insulin resistance, and obesity.^2^ In line, mice on a high-fat diet showed impaired nicotinamide phosphoribosyltransferase (NAMPT)-dependent NAD^+^ biosynthesis in metabolic organs and an adipocyte-specific *Nampt* knockout resulted in severe insulin resistance, which was rescued by NMN administration. Furthermore, NMN improved glucose tolerance in an age-induced T2DM mouse model.^2^ The NAD^+^-consuming enzyme CD38 (cyclic ADP ribose hydrolase) increases with age and plays an important role in age-associated NAD^+^ reduction.^2^ CD38 deficiency improved glucose tolerance, especially when combined with NR supplementation. The specific CD38 inhibitor, 78c, improved age-associated glucose intolerance, muscle function, and exercise capacity.^2^ These and other studies highlight the essential role of NAD^+^ and its metabolites for insulin sensitivity and glucose tolerance in rodents.

Yoshino et al. found that 10 weeks of NMN administration in humans increases muscle insulin sensitivity whereas hepatic and adipose tissue insulin sensitivity, assessed by hyperinsulinemic-euglycemic clamp, remained unaffected.^1^ Muscle NAD^+^ levels of NMN-treated study participants were not altered, but NMN metabolites increased upon NMN treatment, suggesting an increased NAD^+^ turnover (Fig. 1). It has not yet been elucidated if the elevated NAD^+^ usage is responsible for the observed ~25% increase in muscle glucose disposal. To obtain the first mechanistic insights, RNA sequencing was performed on quadriceps muscle samples after NMN supplementation. The platelet-derived growth factor (PDGF) binding pathway was enriched in the treatment group suggesting its involvement in NMN-mediated insulin sensitivity (Fig. 1). The exact underlying molecular mechanism, however, remains unclear and requires further investigation.

In summary, the study by Yoshino et al. provides important first data on the safety and efficacy of NMN supplementation in humans (Fig. 1). The authors compare the NMN-induced improvement in muscle insulin sensitivity with effects induced by (i) the once famous PPARγ agonist troglitazone, which was withdrawn from the market due to safety reasons, and (ii) 10% weight loss.^1^ However, the study was limited to postmenopausal overweight or obese women with prediabetes and, importantly, NMN, in contrast to a significant reduction in body weight, failed to reduce circulating insulin levels or liver fat. Initial studies performed in rodents revealed positive effects of NMN on the insulin response in female mice only.^5^ Moreover, the use of the NAD^+^ precursor NR, instead of NMN as supplementation, did not improve whole-body or muscle insulin sensitivity in other studies with male participants.^1^ Therefore, further clinical studies are required to assess whether NMN functions in a gender-specific way.

Studies in patients with T2DM will show whether NMN is able to decrease the plasma concentrations of glucose and to prevent cardiovascular and renal complications. It will be interesting to compare the effects of NMN to promising new candidates in T2DM treatment such as dual-acting and triple-acting incretin mimetics.^3^ Finally, the safety of long-term NMN application requires further investigation as NAD^+^-depleting drugs showed anti-tumour potential.^2^ Thus, a long-term NMN supplementation might bear the risk of driving tumour growth.
